# Expression of BAFF receptors in muscle tissue of myositis patients with anti-Jo-1 or anti-Ro52/anti-Ro60 autoantibodies

**DOI:** 10.1186/s13075-014-0454-8

**Published:** 2014-10-10

**Authors:** Olga Kryštůfková, Sevim Barbasso Helmers, Paulius Venalis, Vivianne Malmström, Eva Lindroos, Jiří Vencovský, Ingrid E Lundberg

**Affiliations:** Institute of Rheumatology, Na Slupi 4, 128 50 Prague 2, Czech Republic; Department of Rheumatology, First Faculty of Medicine, Charles University in Prague, Kateřinská 32, 121 08 Prague 2, Czech Republic; Rheumatology Unit, Department of Medicine, Karolinska University Hospital in Solna, Karolinska Institutet, Stockholm, Sweden

## Abstract

**Introduction:**

Anti-Jo-1 and anti-Ro52 autoantibodies are common in patients with myositis, but the mechanisms behind their production are not known. Survival of autoantibody-producing cells is dependent on B-cell-activating factor of the tumour necrosis factor family (BAFF). BAFF levels are elevated in serum of anti-Jo-1-positive myositis patients and are influenced by type-I interferon (IFN). IFN-producing cells and BAFF mRNA expression are present in myositis muscle. We investigated expression of the receptors for BAFF in muscle tissue in relation to anti-Jo-1 and anti-Ro52/anti-Ro60 autoantibodies and type-I IFN markers.

**Methods:**

Muscle biopsies from 23 patients with myositis selected based on autoantibody profile and 7 healthy controls were investigated for expression of BAFF receptor (BAFF-R), B-cell maturation antigen (BCMA) and transmembrane activator and calcium modulator and cyclophilin ligand interactor (TACI). Nineteen samples were assessed for plasma (CD138) and B-cell (CD19) markers. The numbers of positive cells per area were compared with the expression of plasmacytoid dendritic cell (pDC) marker blood dendritic cell antigen-2 (BDCA-2) and IFNα/β-inducible myxovirus resistance-1 protein (MX-1).

**Results:**

BAFF-R, BCMA and TACI were expressed in five, seven and seven patients, respectively, and more frequently in anti-Jo-1-positive and/or anti-Ro52/anti-Ro60-positive patients compared to controls and to patients without these autoantibodies (*P* = BAFF-R: 0.007, BCMA: 0.03 and TACI: 0.07). A local association of receptors with B and plasma cells was confirmed by confocal microscopy. The numbers of CD138-positive and BCMA-positive cells were correlated (*r* = 0.79; *P* = 0.001). Expression of BDCA-2 correlated with numbers of CD138-positive cells and marginally with BCMA-positive cells (*r* = 0.54 and 0.42, respectively; *P* = 0.04 and 0.06, respectively). There was a borderline correlation between the numbers of positively stained TACI cells and MX-1 areas (*r* = 0.38, *P* = 0.08).

**Conclusions:**

The expression pattern of receptors for BAFF on B and plasma cells in muscle suggests a local role for BAFF in autoantibody production in muscle tissues of patients with myositis who have anti-Jo-1 or anti-Ro52/anti-Ro60 autoantibodies. BAFF production could be influenced by type-I IFN produced by pDCs. Thus, B-cell-related molecular pathways may participate in the pathogenesis of myositis in this subset of patients.

**Electronic supplementary material:**

The online version of this article (doi:10.1186/s13075-014-0454-8) contains supplementary material, which is available to authorized users.

## Introduction

Idiopathic inflammatory myopathies (IIMs), collectively named *myositis*, are characterised by muscle weakness and inflammation in skeletal muscle tissue. On the basis of their clinical and histopathological features, they may be subclassified into polymyositis (PM), dermatomyositis (DM), immune-mediated necrotising myopathy (IMNM) and inclusion body myositis (IBM). Autoantibodies are frequently found in PM, DM and IMNM, but less often in IBM. Of these, the so-called myositis-specific autoantibodies are closely associated with distinct clinical phenotypes [1]. The most common myositis-specific autoantibodies are the anti-histidyl-tRNA synthetase (anti-Jo-1) autoantibodies, which are present in approximately 10% to 30% of patients with PM and DM [2]. Another subset of autoantibodies is composed of the so-called myositis-associated autoantibodies, of which the anti-Ro52 autoantibodies are the most frequent, being present in 12% to 30% of patients with myositis [3]. These two autoantibodies often co-occur [3,4]. The production site of these autoantibodies is not clear, but B-cell infiltrates and plasma cells have been found in muscle tissue from patients with IIM [5-9] with the presence of clonally related B cells and plasma cells [10] as well as evidence of local affinity maturation [11], which may indicate local autoantibody production in the muscle tissue.

An important factor for B-cell maturation is B-cell-activating factor of the tumour necrosis factor family (BAFF, also known as BLyS) [12]. Elevated serum levels of BAFF have been found in subsets of patients with PM and anti-Jo-1 autoantibodies and in patients with DM [13]. Moreover, serum levels of BAFF correlated positively to serum levels of creatine phosphokinase and negatively to treatment with glucocorticoids, which might indicate that BAFF could have a role in the disease mechanism in subsets of patients with myositis.

BAFF acts through three receptors: BAFF receptor (BAFF-R), B-cell maturation antigen (BCMA) and transmembrane activator and calcium modulator and cyclophilin ligand interactor (TACI). The expression of receptors differs in various stages of B-cell maturation [14,15]. The key receptor for maturation of naïve B cells is BAFF-R, which is expressed by all stages but downregulated during plasma cell differentiation. BCMA is important for the development of mature B cells, is upregulated during differentiation to immunoglobulin (Ig)-secreting cells and supports the survival of long-lived plasma cells in bone marrow [16]. TACI is expressed on activated B cells during differentiation towards plasma cells, provides positive signals to plasmablasts and has a role in isotype switching [17]. However, TACI also negatively regulates clonal expansion of B cells by mechanisms of activation-induced cell death [18], and it could also reduce the availability of BAFF for BAFF-R; hence, TACI has indirect control of BAFF levels [19].

The production of BAFF is induced by type I interferons (IFN-α/β) [20]. In addition, upon IFN-α/β exposure, B cells can differentiate into plasmablasts and increase their resistance to Fas-mediated apoptosis and promote survival [21,22]. The cellular source of type I IFNs, the plasmacytoid dendritic cells (pDCs), together with the IFNα/β-inducible myxovirus resistance 1 (MX-1) protein, has been demonstrated in muscle tissue of patients with PM, DM and IBM [23,24]. Sera from patients with myositis or systemic lupus erythematosus who have anti-Jo-1 and/or anti-Ro52/anti-Ro60 (anti-SSA) autoantibodies have IFN-α-inducing property. It has been suggested that immune complexes containing these autoantibodies directed against RNA-binding proteins may act as endogenous IFN-α/β-inducing factors [23]. Together, these observations may imply that BAFF and type I IFN could interact and play a role in the pathophysiology of myositis with anti-Jo-1 and/or anti-Ro52/anti-Ro60 autoantibodies.

The biological significance of BAFF in the pathogenesis of systemic rheumatic diseases is supported by the high serum levels of BAFF, by expression of BAFF and its receptors in affected organs [25] and by the beneficial effects of neutralisation of BAFF by antibodies (anti-BAFF, anti-BR3) or soluble receptors (TACI-Ig) in model systems and in clinical trials (reviewed in [26]). Blockade of BAFF could also potentially be an alternative to B-cell depletion (by anti-CD20) in patients with myositis [27].

Therefore, the significance of BAFF in myositis needs to be further elucidated. Upregulation of the BAFF mRNA transcript was recently described in muscle tissue of DM, PM and IBM patients [10,28]. Together with the expression of BAFF-R in muscle fibres and infiltrating inflammatory cells, this indicates a potential local function of BAFF in muscle tissue [28]. On the basis of our previous observations of elevated serum levels of BAFF in patients with anti-Jo-1 antibodies [13] and the demonstration of IFN-α-inducing capacity of anti-Jo-1 or anti-Ro52/anti-Ro60-positive sera [23], we postulate that the BAFF pathway could be of particular relevance for a subset of myositis patients with autoantibodies.

The aim of our study was to investigate expression of BAFF receptors (BAFF-R, BCMA and TACI) in the muscle tissues of patients with and those without anti-Jo-1 and/or anti-Ro52/anti-Ro60 autoantibodies, irrespective of the diagnosis DM, PM or IBM. Furthermore, as type I IFNs may induce BAFF production, we aimed to correlate BAFF-R expression in muscle tissue with markers of the type I IFN pathway in muscle tissue samples.

## Methods

### Patients and controls

Muscle biopsy specimens from 23 patients (16 women and 7 men) with or without anti-Jo-1 and/or anti-Ro52/anti-Ro60 autoantibodies according to their medical records were included in this study. They were followed regularly at the Rheumatology Unit, Karolinska University Hospital, Stockholm, and fulfilled the diagnostic criteria for definite or probable PM (*n* =11) or DM (*n* =6) [29,30] or sporadic IBM (*n* =6) [31]. They have been reported previously [23]. The median duration from diagnosis until the time of muscle biopsy was 0.5 years (minumum - maximum range: 0 to 22.5 years), and mean age (SD) was 56.1 ± 12.3 years. At the time of biopsy, 19 patients were being treated with immunosuppressive agents, and the median duration of treatment was 1.6 years (range, 0 to 28.5) (Table 1). Three patients with IBM (patients 10, 17 and 23) (Table 1) formerly diagnosed with PM were treated before the diagnosis of IBM was made (13.7, 9 and 3.2 years, respectively). Biopsy specimens from seven healthy individuals (four women and three men; mean age (SD) =60.7 ± 13.6 years) were included as controls. All patients and control individuals gave their informed consent to participate, and the local ethics committee at the Karolinska Hospital Nord, Stockholm, approved the study.

**Table 1:** **Clinical characteristics and autoantibody profiles of patients at time of muscle biopsy** ^**a**^ **and results of immunohistochemical analysis of biopsies**

**Patient**	**Dg**	**Years since Dg**	**Anti-Jo-1**	**Anti-Ro52**	**Anti-Ro60**	**Other autoantibodies**	**Treatment duration (yr)**	**Treatment at time of biopsy**	**Cumulative clinical manifestations**	Infiltrate score**	**BAFF-R**	**BCMA**	**TACI** ^**b**^	**CD19**	**CD138**	**TACI/vessel** ^**b**^
1	Def PM	−0.8	+	−	−	−	0	No	ILD, A, R	3	+	+	−	+	+	*+*
2	Def PM	0	+	−	−	RNP-70	0	No	ILD	0	-	−	+	−	−	*+*
3	Def PM	0.4	+	−	−	Ku-72	0.3	Yes	ILD, A, R, MH, SS	0	NA	−	−	NA	NA	*−*
4	Def PM	1.6	+	−	−	−	1.6	Yes	ILD, E, T	2	−	−	−	−	+	*+*
5	Prob PM	2.9	+	−	−	−	2.8	Yes	ILD	3	+	+	+	−	+	*+*
6	Prob DM	22.5	+	+	+	La, Ku-86	28.5	Yes	ILD, R, SR	2	−	+	−	−	−	*−*
7	Prob PM	6.5	−	+	+	La	6.4	Yes	A, pSS	3	+	+	+	+	+	*+*
8	Def IBM	0.5	−	+	+	La	0.4	Yes	pSS	3	+	+	+	−	+	*+*
9	Def PM	0.2	−	+	−	−	0.2	Yes	ILD, R	2	−	−	−	NA	NA	*+*
10	Def IBM*	0	−	+	−	−	13.7	Yes	R	2	−	−	+	NA	NA	*+*
11	Def DM	8.8	−	−	−	Mi-2, PM-Scl	9.0	Yes	SR, SS	2	−	−	−	−	+	*+*
12	Def PM	0.8	−	−	−	−	0.5	Yes		0	−	−	−	−	−	*−*
13	Prob PM	−0.1	−	−	−	−	0	No		2	−	−	−	−	+	*+*
14	Prob DM	0	−	−	−	−	0	No	SR	0	−	−	−	−	−	*+*
15	Def PM	5.0	−	−	−	−	4.9	Yes		2	−	−	+	−	+	*−*
16	Def IBM	0.8	−	−	−	Histones	0.8	Yes	E	2	−	+	−	−	+	*+*
17	Def IBM*	0	−	−	−	−	9.0	Yes	R	1	+	−	−	−	+	*+*
18	Def DM	0.3	−	−	−	−	0.3	Yes	SR	1	−	−	−	NA	NA	*+*
19	Prob DM	0.1	−	−	−	−	0.2	Yes	SR	2	−	+	−	NA	NA	*+*
20	Def IBM	8.4	−	−	−	−	8.4	Yes		0	−	−	−	NA	NA	*+*
21	Def PM	9.4	−	−	−	−	9.7	Yes	A	0	−	−	−	NA	NA	*+*
22	Def DM	13.2	−	−	−	Ku-86	13.2	Yes	SR	1	−	−	−	−	−	*−*
23	Prob IBM*	−1.1	−	−	−	−	2.1	Yes		0	−	−	+	NA	NA	*+*

^a^A, Arthritis; BAFF-R, B-cell-activating factor of the tumour necrosis factor family receptor; BCMA, B-cell maturation antigen; Def, Definitive; Dg, Diagnosis; DM, Dermatomyositis; E, oesophagus; IBM, Inclusion body myositis; ILD, Interstitial lung disease; MH, Mechanic’s hands; NA, Not assessed; PM, Polymyositis; Prob, Probable; pSS, Primary Sjögren’s syndrome; R, Raynaud’s phenomenon; SR, Skin rash; SS, Sjögren’s syndrome; T, Thrombosis; TACI, Transmembrane activator and calcium modulator and cyclophilin ligand interactor. ^b^TACI was also expressed in vessels (*) at time of biopsy diagnosed as PM, (**) score: 0=no infiltrate; 1=scattered cells; 2=one or two small infiltrates; 3=several small or one big infiltrate or infiltrate + scattered cells.

### Autoantibody detection

Line blot assays (Inno-Lia ANA update test; Innogenetics, Ghent, Belgium; and Myositis LIA; IMTEC Immundiagnostika, Berlin, Germany) and Western blot assay (anti-myositis antigen EUROLINE-WB kit; Euroimmun, Lubeck, Germany) were used to define autoantibody profiles as previously described [13]. The tests included specificities of autoantibodies against the following antigens: Jo-1/HRS, SmB, SmD, ribonucleoprotein (RNP)-70K, RNP-A, RNP-C, Ro52/SSA, Ro60/SSA, La/SSB, centromere B, topoisomerase-1/Scl70, ribosomal P antigen, histones, Mi-2, PM-Scl/100, U1-snRNP, Ku72/86, PL-7 and PL-12. Patients who had negative results for anti-Jo-1 and anti-Ro52/anti-Ro60 in all three specific tests were considered not to have these autoantibodies.

### Muscle biopsy specimens

Muscle tissue specimens were obtained by using a semiopen technique while the patients were under local anaesthesia [32]. The 7-μm-thick serial cryostat sections were mounted on gelatin-coated glass slides (Cell-Line Associates, Newfield, NJ, USA) and fixed in 2% formaldehyde as previously described [23]. The serial sections were stored at −80°C until stained. To reduce the possibility of selection bias, the first and last muscle tissue sections of a series of specimens were stained with haematoxylin and eosin to detect inflammatory cell infiltrates and to exclude any inconsistencies in the biopsies. Usually, two biopsies were sectioned and similar results were obtained.

### Immunohistochemistry

The staining procedure applied was a peroxidase method previously described [23], with some modifications, as we used endogenous peroxidase block (with 1% H_2_O_2_ and 2% NaN_3_), avidin-biotin (Vector Laboratories, Burlingame, CA, USA) and 1% normal horse serum in blocking steps. Phosphate-buffered saline (PBS)-saponin (0.1%) was used for washing and antibody dilution.

Sections were stained overnight using primary antibodies towards receptors for BAFF (BAFF-R, clone 265.13.1.9.3.2, final dilution 1 μg/ml), BCMA (clone 255.4.1.2.1.5, final dilution 4 μg/ml) and TACI (clone 250.13.1.1.4.3, final dilution 2 μg/ml), all kindly provided by ZymoGenetics (Seattle, WA, USA). From 15 of the patients and from 4 of the controls, muscle tissue was available for staining for B cells using antibodies to CD19 (clone HD37, 0.6 μg/ml) and for plasma cells using anti-CD138 (clone M115, 1.8 μg/ml) (both from Dako, Glostrup, Denmark). All primary antibodies used were mouse anti-human IgG1 monoclonal antibodies. Isotype-matched irrelevant antibody (mouse IgG_1_κ, clone DAK-G01 in 4 μg/ml dilution; Dako) was used as the control.

Following washing and staining steps (as described previously [23]) with secondary biotinylated horse anti-mouse IgG antibodies (Vector Laboratories) diluted 1:320 in PBS-saponin with 1% horse serum, incubation with avidin-biotin-horseradish peroxidase complex (Vectastain ABC Elite Kit; Vector Laboratories), development with 3,3′-diaminobenzidine (DAB substrate kit; Vector Laboratories) and a final counterstaining step with Mayer’s haematoxylin were performed. Sections from human tonsils were used for titration of primary antibodies and as positive controls for staining of B cells, plasma cells and receptors for BAFF (Additional file 1: Figure S1). For expression of pDC marker blood dendritic cell antigen 2 (BDCA-2/CD303) and MX-1 protein, we used already stained slides where consecutive serial sections were available from the same patients (*n* =23) [23].

### Evaluation of immunohistochemical staining

Entire tissue sections were analysed using a conventional microscope (Reichert-Jung Polyvar 2; Leica, Vienna, Austria) in a coded manner by three independent assessors. The mean numbers of cells positive for receptors for BAFF, CD19 and CD138 per square millimetre of muscle tissue were calculated. The number of cells positive for staining for receptors was tested for a possible correlation with the expression of pDC marker BDCA-2/CD303 and MX-1 protein in consecutive serial sections, expressed as the percentage of positively stained area per total tissue section (Table 2). A quantitative evaluation of BDCA-2 and MX-1 protein expression was performed by computerised image analysis on the total tissue area using the Leica QWin software and microscope (DM RXA2; Leica, Wetzlar, Germany). There was a high degree of correlation between the results of conventional microscopic evaluation and those of computerised image analysis of BDCA-2-positive cells and of total MX-1 expression, as previously reported [23].

**Table 2:** **Correlations between expression of receptors for BAFF and expression of markers for B and plasma cells, type I IFN production and plasmacytoid dendritic cells in muscle tissue** ^**a**^

	**BAFF-R** ^**b**^	**BCMA** ^**b**^	**TACI** ^**b**^	**CD19** ^**b**^	**CD138** ^**b**^
	**(** ***n*** **=15)**	**(** ***n*** **=15)**	**(** ***n*** **=15)**	**(** ***n*** **=12)**	**(** ***n*** **=12)**
CD19^b^	0.49^†^	0.44^†^	0.19		
(*n* =12)					
CD138^b^	0.70**	0.79***	0.39	0.39	
(*n* =12)					
MX-1^c^	0.23	0.33	0.38^†^	0.12	−0.01
(*n* =15)					
BDCA-2^c^	0.16	0.42^†^	0.23	0.37	0.54*
(*n* =15)					

^a^BAFF-R, B-cell-activating factor of the tumour necrosis factor family receptor; BCMA, B-cell maturation antigen; BDCA-2, Blood dendritic cell antigen 2; MX-1, Interferon α/β–inducible myxovirus resistance 1 protein; TACI, Transmembrane activator and calcium modulator and cyclophilin ligand interactor. Data included to correlation analysis were: ^b^Numbers of positive cells counted by conventional microscopy per area (*n*/mm^2^). Included samples which expressed at least one of the receptors. ^c^Quantitative evaluation of expression by computerised image analysis (percentage of positively stained area). Data are correlation coefficients of Spearman´s rank order test. ***P <0.005, **P <0.01, *P <0.05, ^†^ *P* <0.1.

### Immunofluorescent staining and confocal microscopy

Muscle tissue sections were fixed in 2% formaldehyde PBS and washed in PBS. Saponin 0.1% PBS solution was used for permeabilisation, blocking solutions and washing. Serum blocking was performed with 4% normal human serum (NHS) and 6% animal serum (same species as secondary antibody). A kit was used for avidin-biotin blocking (Vector Laboratories). Antibodies were diluted in PBS-saponin with 1% NHS.

Sections were stained overnight at 4°C with primary antibodies towards receptors for BAFF (1:400 dilution) as described above and with goat polyclonal antibodies to CD19 (1:400 dilution; Santa Cruz Biotechnology, Inc, Dallas, Texas, USA) or anti-CD138 (1:400 dilution; R&D Systems Europe, Abingdon, UK). Samples were incubated for 1 hour with secondary biotinylated horse anti-mouse IgG antibodies (1:300 dilution; Vector Laboratories) and donkey anti-goat polyclonal antibodies (1:300 dilution, Alexa Fluor 594 conjugate; Life Technologies, Carlsbad, CA, USA), followed by incubation with streptavidin-Alexa Fluor 488 conjugate (1:300 dilution; Molecular Probes, Eugene, OR, USA). 4′,6-diamidino-2-phenylindole was used to visualise the nuclei. The Leica QWin software and microscope were used to detect the immunofluorescent staining and for confocal microscopy.

### Statistical analyses

Data were analysed using GraphPad Prism statistical software (version 5.02; GraphPad Software, La Jolla, CA, USA). For analysis of differences between groups, a Kruskal-Wallis with Dunn’s *post hoc* test and a nonparametric Mann-Whitney *U* test were performed. Spearman’s rank-order test was used for correlations of parameters. A *P*-value ≤0.05 was considered statistically significant. Data are presented as median and range from minimum to maximum or mean ± SD based on distribution.

## Results

### Clinical, laboratory and serologic findings

Ten patients were positive and thirteen patients were negative for anti-Jo-1 or anti-Ro52/anti-Ro60 autoantibodies and constituted the two groups whose muscle tissue was analysed. Detailed autoantibody profiles are presented in Table 1.

### BAFF-R, BCMA and TACI expression in muscle tissue

**Figure 1:**
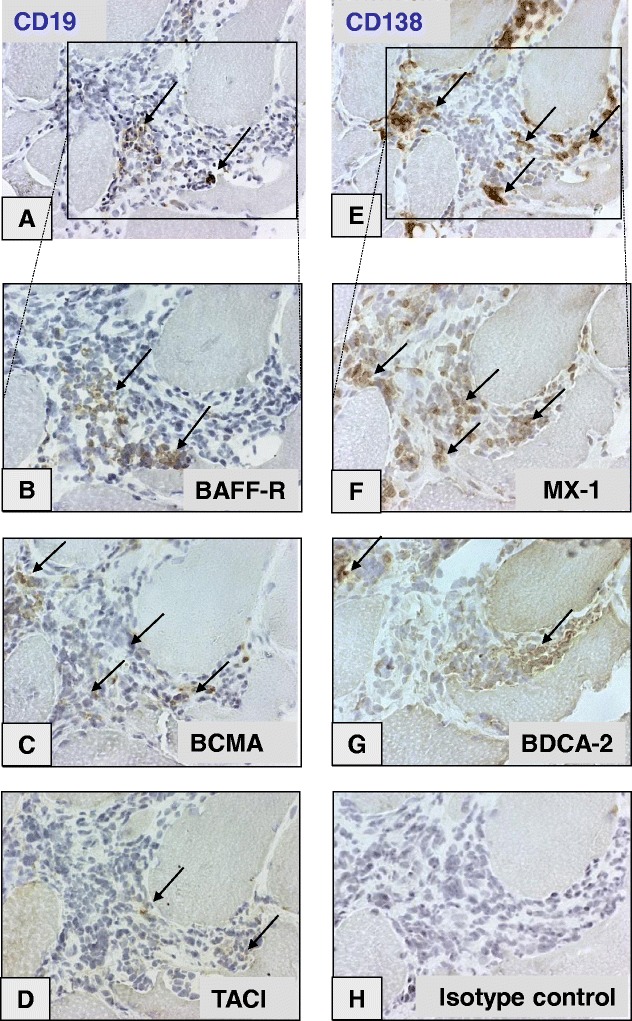
**Serial sections of muscle tissue mononuclear cell infiltrate.** These sections were taken from a representative anti-Ro52/anti-Ro60-positive patient with polymyositis. They are immunohistochemically stained for B-cell marker CD19 **(A)** and plasma cell marker CD138 **(E)**. Original magnification, ×25. Details of infiltrate (original magnification, 1.25 × 25) stained for B cell–activating factor of the tumour necrosis factor family receptor (BAFF-R) **(B)**, B cell maturation antigen (BCMA) **(C)**, transmembrane activator and calcium modulator and cyclophilin ligand interactor (TACI) **(D)**, interferon α/β–inducible myxovirus resistance 1 (MX-1) protein **(F)** and plasmacytoid dendritic cell marker blood dendritic cell antigen 2 (BDCA-2) **(G)** compared to immunoglobulin G1 isotype control **(H)**. Brown colour indicates positively stained cells, indicated by arrows.

Mononuclear cell infiltrates were present in 16 of 23 patient samples (Table 1). In 11 of them, one or more receptors for BAFF were expressed. BAFF-R, BCMA and TACI expression was present in five, seven and seven of the eleven positive samples. In addition, scattered TACI-positive cells were seen in biopsies from two patients without infiltrates. BAFF-R positivity (median, 1.8 cells/mm^2^ (minimum 0.5 to maximum 55.0)) was seen in clusters of infiltrating mononuclear cells, which included CD19-positive B cells (Figures 1A and 1B, Additional file 2: Figures S2A and S2B). The receptors BCMA (3.2 cells/mm^2^ (0.14 to 11.2)) and TACI (1.5 cells/mm^2^ (0.3 to 16.6)) were observed both in mononuclear inflammatory cell infiltrates (Figures 1C and 1D, Additional file 3: Figure S3A) and within the muscle tissue in scattered cells with plasma cell–like morphology. Blood vessels expressing TACI in endothelial cells or smooth muscle tissues were present in 18 patients (Table 1) and 5 healthy individuals. Occasional TACI-positive cells were recorded in three of the healthy control biopsies, but BAFF-R and BCMA staining was negative in all controls.

Taken together, all three receptors were expressed in cells located in inflammatory infiltrates. Of the five tissue samples with expression of BAFF-R in inflammatory infiltrates, BCMA was expressed in four and TACI in three.

The number of cells per square millimetre expressing BAFF-R correlated with the number of cells per square millimetre expressing BCMA (n = 8, *r*_S_ = 0.76, *P* = 0.02). Of the seven BCMA-positive samples, three were also positive for TACI.

The three receptors were distributed into different sublocalisations of the infiltrates. BAFF-R- and BCMA-expressing cells were localised in separate clusters within infiltrates (Figures 1B and 1C), whereas TACI was expressed mainly by scattered mononuclear cells of plasma cell–like morphology and in areas positive for plasma cell marker CD138 in the endomysium (Additional file 3: Figures S3A and S3B). Within infiltrates, BAFF-R expression was observed in two patients in large clusters of cells, many of which were CD19-positive (up to 100 cells) (Additional file 2: Figure S2B), whereas BCMA was present in other and smaller clusters (approximately 10 cells) (Figure 1C) and TACI was weakly positive in scattered cells within infiltrates (Figure 1D).

### B cells and plasma cells in muscle tissue and colocalisation with BAFF receptors and with plasmacytoid dendritic cell markers

Consecutive sections of muscle biopsies from 15 patients were available for staining of B cell and plasma cell markers. Plasma cells (CD138-positive) were detected in 10 patients (4.7 cells/mm^2^ (1.1 to 161.7)) within cellular infiltrates (Figure 1E) or as scattered cells in endomysial and/or perivascular areas. Accumulations of CD19-positive B cells were observed in only two patients (7.1 and 9.1 cells/mm^2^), both in large mononuclear infiltrates (Figure 1A, Additional file 2: Figure S2A).

**Figure 2:**
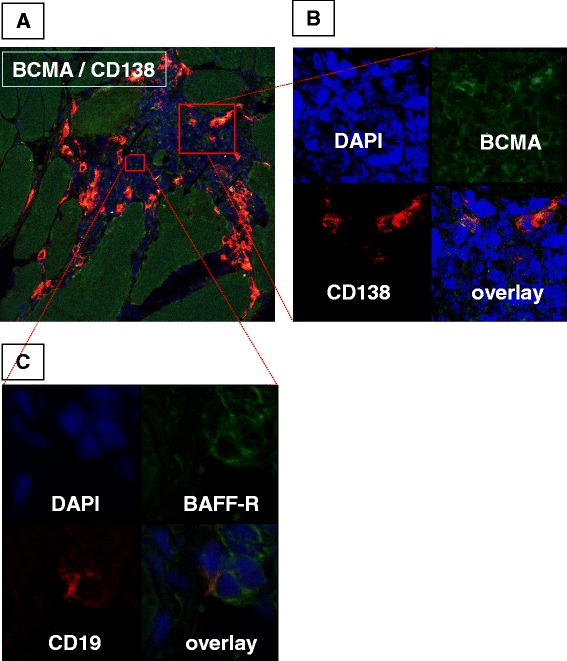
**Immunofluorescent double-staining of B and plasma cells expressing receptors for B cell–activating factor of the tumour necrosis factor family in the muscle mononuclear cell infiltrate from a representative anti-Ro52/anti-60-positive patient with polymyositis.** Immunofluorescence staining for plasma cell marker CD138 (red) and B cell maturation antigen (BCMA) (green) **(A)** in a tissue biopsy section from the same patient as in Figure 1 (original magnification, ×200) with details **(B)** of staining for CD138, BCMA, 4′,6-diamidino-2-phenylindole (DAPI) and an overlay created with the confocal microscope (original magnification, ×600). Scattered CD19-positive cells were also present remote from the CD138-positive cells and were positive for BAFF-R **(C)** as expressed in details of CD19 (red), BAFF-R (green), DAPI (blue) and an overlay created with the confocal microscope (original magnification, ×600).

The expression pattern in serial sections suggests that CD138-positive plasma cells colocalised with the distribution of BCMA-positive cells (Figures 1C and 1E) and that some plasma cells also expressed TACI, albeit weakly (Figures 1D and 1E, Additional file 3: Figures S3A and S3B). BAFF-R positivity was seen in cells in the same areas as CD19-positive B cells in consecutive sections (Figures 1A and 1B, Additional file 2: Figures S2A and S2B). The number of CD138-expressing cells correlated positively with the number of BCMA-expressing cells (*r*_S_ = 0.79, *P* = 0.001) and also with BAFF-R (*r*_S_ = 0.70, *P* = 0.006) (Table 2). Colocalisation of CD138 and BCMA was confirmed by confocal microscopy with CD138/BCMA double-positive cells in serial consecutive sections, within infiltrates (Figures 2A and 2B) and as scattered infiltrating cells within muscle tissue.

BAFF-R- and CD138-positive cells were distributed in different sublocalisations of tissue infiltrates. Therefore, double-staining for these two markers was not done. In both patients with CD19-positive B cells, the CD19/BAFF-R double-positive clusters were present within mononuclear infiltrates (Figure 2C and Additional file 2: Figures S2C and S2D). In two other samples (Table 1, patients 5 and 8), several BAFF-R single-positive cells were detected by light and immunofluorescence microscopy. The number of CD19-expressing cells weakly correlated with expression of BAFF-R (*r*_S_ = 0.49, *P* = 0.05) and BCMA (*r*_S_ = 0.44, *P* = 0.08) (Table 2).

**Figure 3:**
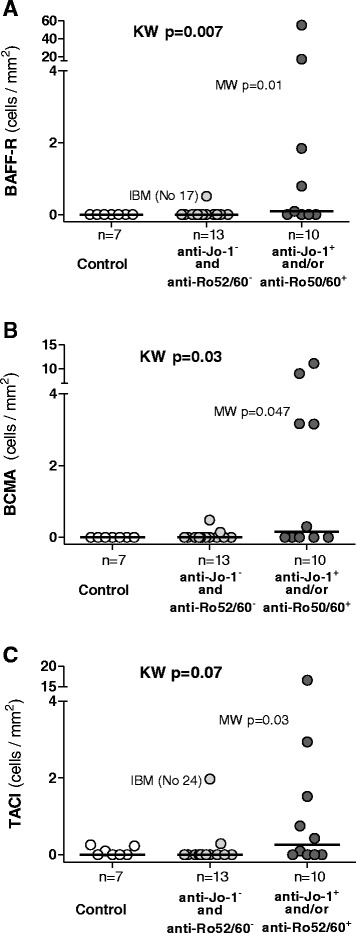
**Quantitative assessment of the expression of receptors for B cell–activating factor of the tumour necrosis factor family in muscle tissues.** Cells positive for B cell–activating factor of the tumour necrosis factor family receptor (BAFF-R) **(A)**, B cell maturation antigen (BCMA) **(B)** or transmembrane activator and calcium modulator and cyclophilin ligand interactor (TACI) **(C)** per square millimetre of muscle tissue area are shown for healthy controls and patients with myositis with or without anti-Jo-1 and/or anti-Ro52/anti-Ro60 autoantibodies. IBM, Inclusion body myositis. Data presented are *P-*values calculated by Kruskal-Wallis (KW) test and Mann-Whitney (MW) *U* test. Horizontal bars represent medians for each group.

In the same areas of infiltrates with CD138-positive plasma cell expression, we found cells positively stained for MX-1 protein (Figure 1F) and BDCA-2 (that is, pDCs) (Figure 1G and Additional file 3: Figure S3D). The percentage area of BDCA-2-positive pDCs, as estimated by computerised image analysis, correlated with the number of cells expressing CD138 (*r*_S_ = 0.54, *P* = 0.04) (Table 2) and weakly with BCMA (*r*_S_ = 0.42, *P* = 0.06) (Table 2, Figure 3) [23]. In addition, there was a weak correlation between number of TACI-positive cells and MX-1-positive cells (*r*_S_ = 0.38, *P* = 0.08) (Table 2).

### BAFF-R, BCMA and TACI expression in muscle tissue in relation to autoantibody profile and clinical manifestations

The number of BAFF-R- and BCMA-expressing cells per area was higher in biopsies from patients with anti-Jo-1 and/or anti-Ro52/anti-Ro60 autoantibodies compared to patients without these autoantibodies and to healthy controls (*P* = 0.007 and *P* = 0.03, respectively, by Kruskal-Wallis test; with Dunn’s *post hoc* test, both *P* <0.05). The Mann-Whitney *U* test results showed significant differences in expression of BAFF-R, BCMA and TACI when we compared patients with versus without anti-Jo-1 or anti-Ro52/anti-Ro60 autoantibodies (*P* = 0.01, 0.047 and 0.03, respectively) (Figure 3).

No significant differences in expression of BAFF-Rs or the presence of B cells or plasma cells in muscle tissue were recorded with respect to PM, DM or IBM subdiagnoses. There were no associations between BAFF-Rs or B and plasma cell markers and disease or treatment duration, or with treatment at time of biopsy (Table 1).

## Discussion

A major finding in our study is the expression of receptors for BAFF in mononuclear cells in muscle tissue of patients with myositis. The presence of the receptors was associated with detectable plasma cells, as well as with the presence of anti-Jo-1 and anti-Ro52/anti-Ro60 autoantibodies in sera. In addition, there was a correlation between number of cells expressing receptors for BAFF, number of plasma cells and expression of markers of pDCs. These data are suggestive of local BAFF-driven differentiation of autoantibody-producing plasma cells in muscle tissue in patients with myositis who are seropositive for anti-Jo-1 or anti-Ro52/anti-Ro60 autoantibodies.

Our data, in addition to the already described beneficial effects of B lymphocyte depletion by rituximab (anti-CD20) in refractory PM or DM [27,33-35], including patients with antisynthetase syndrome [27,33], support a role for local autoreactive B lymphocytes in these conditions. A correlation between serum levels of anti-Jo-1 autoantibodies and disease activity in patients with myositis has been described [36], but it is not known where the anti-Jo-1 autoantibodies are produced. The target antigen for anti-Jo-1 autoantibodies—histidyl tRNA synthetase—is ubiquitously expressed, but, interestingly, with higher expression in epithelial cells of lungs compared to other healthy organs, and also in higher extent in regenerating muscle fibres than in differentiated fibres [37]. Therefore, a local autoantigen presentation in muscle tissue could be considered.

**Figure 4:**
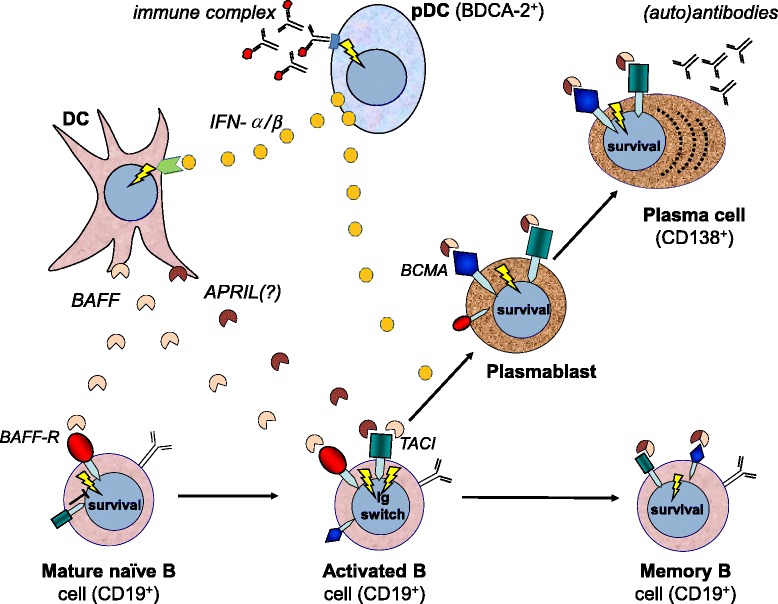
**Schematic illustration of suggested interaction of dendritic cells, type I interferon and local B cell differentiation in myositis muscle.** Plasmacytoid dendritic cells (pDCs) stimulated by immunocomplexes produce type I interferon (IFN-α/β) and induce conventional dendritic cells (DCs) to secrete the cytokines B cell–activating factor of the tumour necrosis factor family (BAFF) and a proliferation-inducing ligand (APRIL), which influence B cell differentiation and survival. Their three receptors—BAFF receptor (BAFF-R), B cell maturation antigen (BCMA) and transmembrane activator and calcium modulator and cyclophilin ligand interactor (TACI)—are differentially expressed during the maturation of B cells from naïve to memory B cells and plasma cell generation. Class switch, differentiation to plasmablast and survival are also enhanced by IFN-α/β. The presence of APRIL in myositis muscle is unclear, but its importance for immunoglobulin (Ig) class switch is known.

In our patients, we saw only modest numbers of CD19-positive B cells in muscle tissue, whereas the presence of CD138-positive plasma cells was more prominent. This may imply that B cells continue to differentiate in this environment, which is supported by the beautiful sequence analysis by Salajegheh *et al*. [10]. Also, in other studies, researchers have demonstrated this unusual bias for plasma cells [9,11]. A similar low level expression of CD19 on long-lived plasma cell precursors has been described in peripheral blood from patients with primary Sjögren’s syndrome, rheumatoid arthritis and active systemic lupus erythematosus [38-40], and downregulation of CD19 expression during differentiation into antibody-secreting cells has been documented [41,42]. It is tempting to speculate that, similarly to the kidneys of patients with lupus nephritis, affected muscles of patients with IIM could provide a survival niche for autoantibody-producing plasma cells [43,44]. Our hypothesis is that the maturation of plasma cells and possible affinity maturation within muscle tissue of patients with myositis could be driven by the autoantigen in muscle tissue under the permissive cytokine environment (Figure 4).

In another study, which included patients with dermatomyositis, a significant increase in mRNA levels of BAFF, but not BAFF-R, was reported in muscle tissue compared to normal controls [28]. Using a polyclonal anti-BAFF-R antibody, the authors described strong staining of BAFF-R on some inflammatory cells. However, they also found BAFF and BAFF-R on regenerating and necrotic muscle fibres, but not in normal muscle. We could not confirm the staining for BAFF-R in regenerative or necrotic fibres, because the BAFF-R expression was limited to inflammatory cells. The discrepancy between the staining patterns in these two studies can probably be explained by different specificities of antibodies used for staining, whereas monoclonal antibodies, as used in our study, usually are more specific. Furthermore, the staining pattern was validated on tonsil tissue (Additional file 1: Figure S1), where we observed BAFF-R positivity in areas positive for CD19 cells with patterns similar to those described for expression in tonsillar [15] and reactive lymphoid [45] tissues.

The number of cells expressing BAFF-R, BCMA or TACI was significantly higher in patients with anti-Jo-1 and anti-Ro52/anti-Ro60 autoantibodies compared to patients without these autoantibodies. Moreover, the receptors were localised to areas with B cells and plasma cells on serial sections. The double-positivity of receptors and B cells or plasma cells, as suspected on the basis of the distribution of cells expressing BAFF-R or BCMA, was confirmed by immunofluorescent staining and confocal microscopy. This is consistent with the known differential expression of receptors for BAFF during the development of B cells into Ig-producing plasma cells [14,15].

The number of cells expressing CD138 correlated positively with expression of BAFF-R. This was a statistical correlation, but the cells were present in separate areas within the infiltrates. The correlation between BAFF-R and CD138 may reflect the presence of activated BAFF-R-positive T cells, supporting a differentiation of B cells into plasma cells in addition to the BAFF/APRIL and IFN signals. This remains to be proven because the staining for T cell markers was outside the scope of this study.

Two or more receptors for BAFF appeared together in several tissue samples, but they were distributed into different sublocalisations of the infiltrates or in scattered cells. The numerical correlation between the number of cells expressing BAFF-R and the number of BCMA-positive cells could be explained by the presence of B cells in various differentiation stages in individual tissues.

The finding of plasma cells in areas with plasmacytoid DC markers, together with the receptor BCMA on serial sections and the trend for quantitative correlations between these markers, suggests a local role for pDCs in inducing differentiation of autoantibody-producing cells in muscle tissue. This is further supported by the presence of MX-1 in areas with plasmacytoid DCs and plasma cell markers. The pDCs represent a local source of type I IFN and have previously been described in inflamed myositis muscle tissue [23,24]. Excitingly, B cells can enhance IFNα production by pDCs [46], which in turn could locally stimulate myeloid DCs (shown in PM and IBM muscle [47]) to produce BAFF (as reviewed previously [48]). Plasma cell survival and antibody secretion are known to be enhanced by BAFF [49], and local affinity maturation of B lymphocytes and plasma cells in muscle tissue of patients with myositis has been reported [9-11]. During B cell differentiation to plasma cells, BAFF-R expression is downregulated and BCMA is upregulated, which results in differential local expression of these receptors in muscle tissue infiltrates. The possible role of locally produced type I IFN is also consistent with our previous findings of a negative correlation between serum levels of IFN-α with muscle involvement as assessed by magnetic resonance imaging [50].

There are some limitations of this study. One is the weak staining for TACI. Therefore, we may have underestimated the TACI expression. However, the TACI staining was distinct from the negative isotype control, and the concentration of the antibody was chosen with respect to background staining in muscle fibres. We could see expression of TACI in scattered cells of patient samples without infiltrates and also in three control tissues. This could be related to TACI staining of vessels, which has also been reported in other tissues [51]. This uncertainty could modify the correlation between TACI-positive cells and cells expressing CD138 and BDCA-2, which was weak. Expression of TACI on DCs has been demonstrated on *in vitro* monocyte-derived DCs [52]. Indeed, we observed TACI expression in BDCA-2-positive areas (Figure 2 and Additional file 3: Figure S3). The limitation of the availability of muscle tissue explains why we had samples available for detecting B cell markers from only 15 of the 23 patients.

There were some patients with anti-Jo-1 or anti-Ro52/anti-Ro60 autoantibodies without BAFF-R expression in muscle tissue. Importantly, these patients did not have detectable inflammatory cell infiltrates in either the first or last section of a series, which reflects the patchy nature of the inflammatory infiltrates often observed in muscle biopsies from patients with myositis.

## Conclusions

Our data indicate a possible local role for BAFF in muscle tissue from patients with myositis, particularly in a subset of patients with anti-Jo-1 or anti-Ro52/anti-Ro60 autoantibodies. The presence of receptors for BAFF in the vicinity of, and even colocalised with, B cells and plasma cells further substantiates the possibility that local autoantibody production could occur in the muscle and might be induced by type I IFN produced by pDCs. BAFF may thus be a potential target for treatment in this subset of patients with myositis.

## Additional files

Additional file 1: Figure S1. Immunohistochemistry staining for plasma cell marker (CD138), B-cell marker (CD19) in human tonsil. Serial sections stained for receptors for BAFF (BCMA, TACI and BAFF-R). Brown colour indicates positively stained cells. BAFF-R is present in the areas with B cells, while BCMA and TACI were expressed in the plasma cell (CD138) positive areas. Upper left panel shows staining with IgG1 isotype control (original magnification, ×25; detail magnification, 1.25 × 25). Additional file 2: Figure S2. Immunohistochemistry staining for B cell marker CD19 **(A)** and BAFF-R **(B)** in an infiltrate of muscle tissue from representative anti-Jo-1-positive patient with polymyositis. Brown colour indicates positively stained cells (original magnification, ×250). The weak expression of CD19 could reflect the differentiation of B cells into the pre-plasma cells. Double-positivity for B cell marker CD19 and BAFF-R **(C)** seen by fluorescent microscopy (original magnification × 200), with details of staining for CD19 (red), BAFF-R (green), DAPI (blue) and overlay **(D)** from confocal microscope (original magnification, ×600). Additional file 3: Figure S3. Immunohistochemistry staining for TACI **(A)**, plasma cell marker CD138 **(B)**, isotype control **(C)** and a marker of plasmacytoid dendritic cell BDCA-2 **(D)** in an endomysial infiltrate of patient with polymyositis. Brown colour indicates positively stained cells (original magnification, ×250). Details of staining for CD138 (red), TACI (green), DAPI (blue), overlay **(E)** and enlargement of double positivity for plasma cell marker CD138 and TACI **(F)** seen by confocal microscope (original magnification, ×600).

## Abbreviations

A: Arthritis
BAFF: B cell–activating factor of the tumour necrosis factor family
BAFF-R: BAFF receptor
BCMA: B cell maturation antigen
BDCA-2: Blood dendritic cell antigen 2
def: Definitive
Dg: Diagnosis
DM: Dermatomyositis
F: Female
IBM: Inclusion body myositis
ILD: Interstitial lung disease
M: Male
MH: Mechanic’s hands
MX-1: IFNα/β-inducible myxovirus resistance 1 protein
NA: Not assessed
pDC: Plasmacytoid dendritic cell
PM: Polymyositis
prob: Probable
pSS: Primary Sjögren’s syndrome
R: Raynaud
SR: Skin rash
SS: Sjögren’s syndrome
T: Thrombosis
TACI: Transmembrane activator and calcium modulator and cyclophilin ligand interactor
