# Supplementary figures and images for: Expression of BAFF receptors in muscle tissue of myositis patients with anti-Jo-1 or anti-Ro52/anti-Ro60 autoantibodies
Source: Arthritis Res Ther. 2014 Oct 10;16(5):454. doi: 10.1186/s13075-014-0454-8 (PMC4234835; doi:10.1186/s13075-014-0454-8)

Additional figure 1

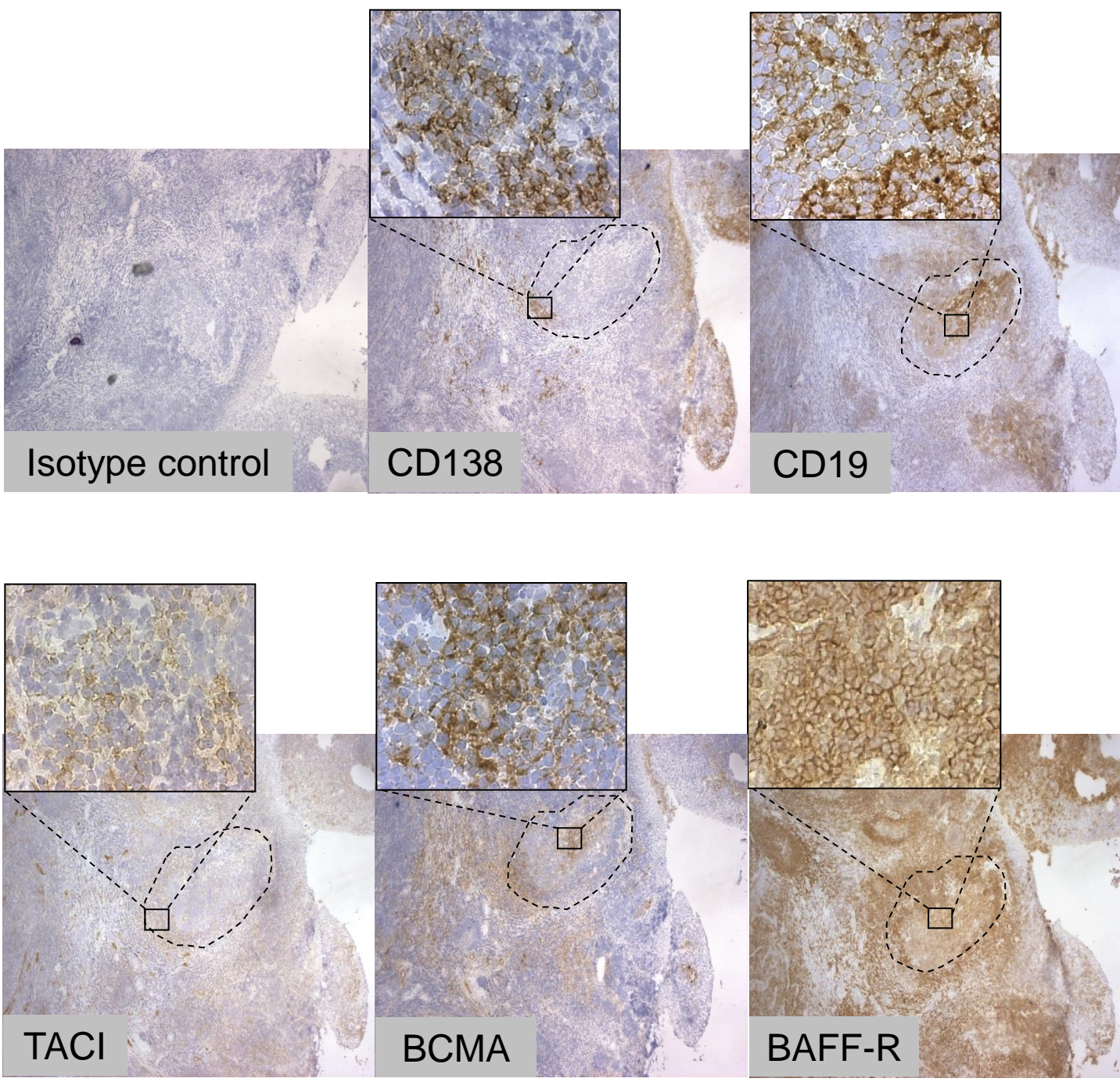

Supplement: Additional file 1: Figure S1. — Immunohistochemistry staining for plasma cell marker (CD138), B-cell marker (CD19) in human tonsil. Serial sections stained for receptors for BAFF (BCMA, TACI and BAFF-R). Brown colour indicates positively stained cells. BAFF-R is present in the areas with B cells, while BCMA and TACI were expressed in the plasma cell (CD138) positive areas. Upper left panel shows staining with IgG1 isotype control (original magnification, ×25; detail magnification, 1.25 × 25). [file 13075_2014_454_MOESM1_ESM.pdf]

## Additional figure 2

**A**

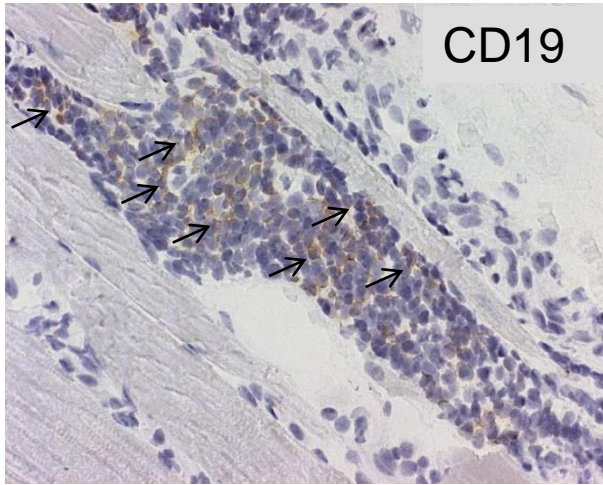

**B**

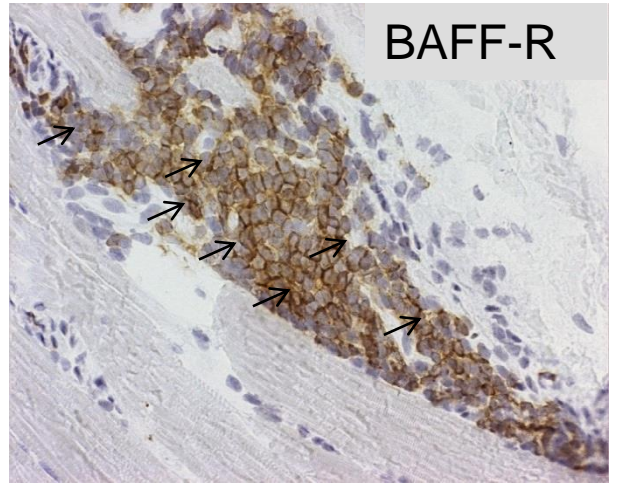

**C**

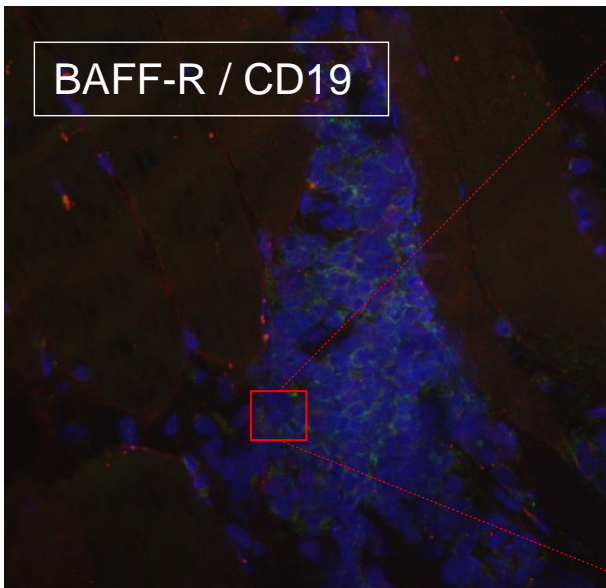

**D**

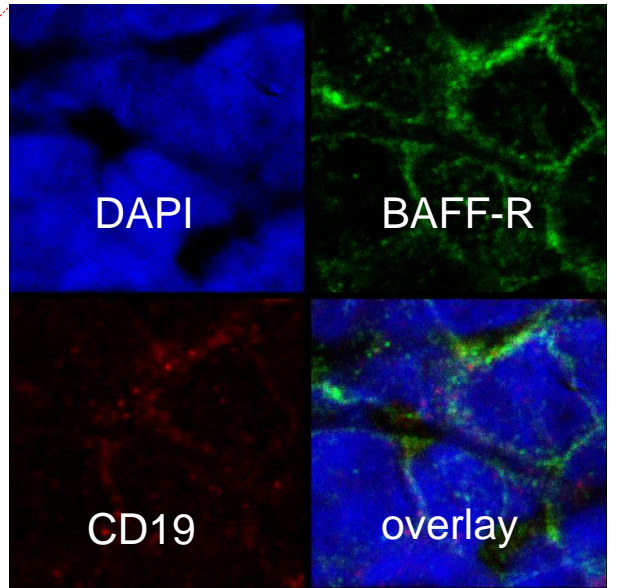

Supplement: Additional file 2: Figure S2. — Immunohistochemistry staining for B cell marker CD19 (A) and BAFF-R (B) in an infiltrate of muscle tissue from representative anti-Jo-1-positive patient with polymyositis. Brown colour indicates positively stained cells (original magnification, ×250). The weak expression of CD19 could reflect the differentiation of B cells into the pre-plasma cells. Double-positivity for B cell marker CD19 and BAFF-R (C) seen by fluorescent microscopy (original magnification × 200), with details of staining for CD19 (red), BAFF-R (green), DAPI (blue) and overlay (D) from confocal microscope (original magnification, ×600). [file 13075_2014_454_MOESM2_ESM.pdf]

### Additional figure 3

**A**

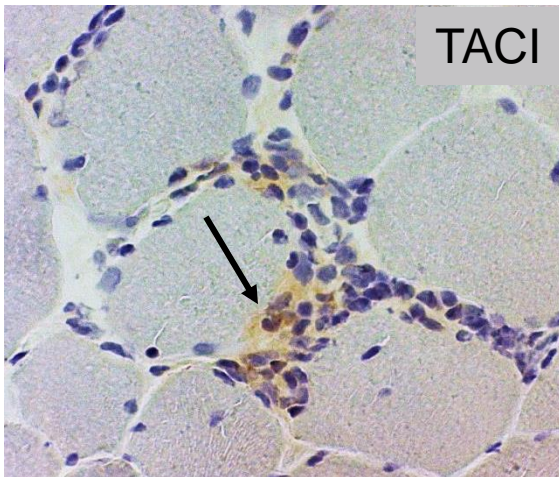

**B**

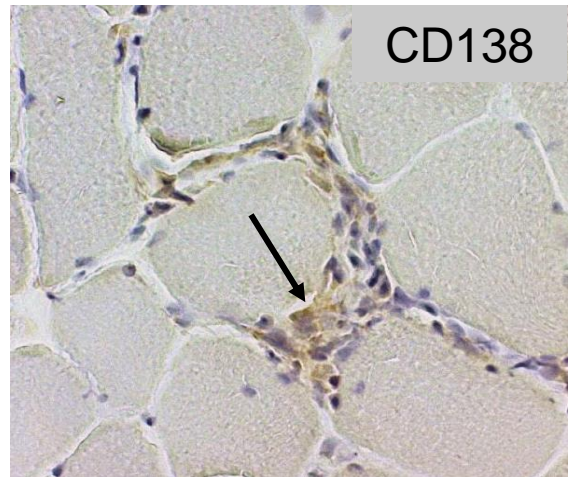

**C**

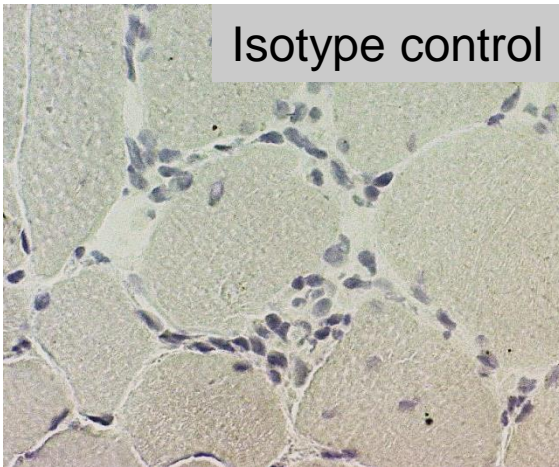

**D**

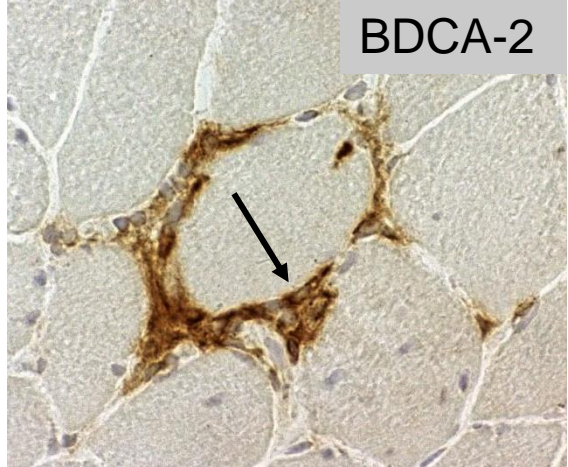

**E**

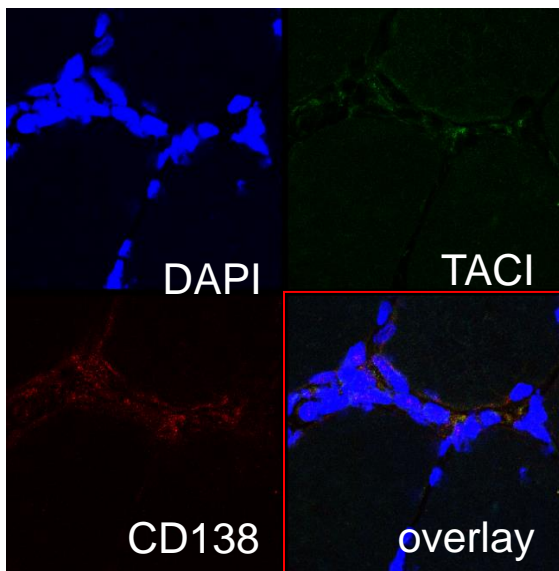

**F**

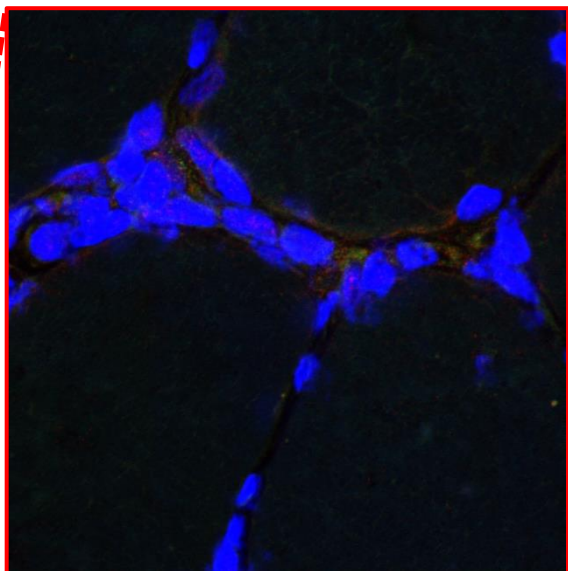

Supplement: Additional file 3: Figure S3. — Immunohistochemistry staining for TACI (A), plasma cell marker CD138 (B), isotype control (C) and a marker of plasmacytoid dendritic cell BDCA-2 (D) in an endomysial infiltrate of patient with polymyositis. Brown colour indicates positively stained cells (original magnification, ×250). Details of staining for CD138 (red), TACI (green), DAPI (blue), overlay (E) and enlargement of double positivity for plasma cell marker CD138 and TACI (F) seen by confocal microscope (original magnification, ×600). [file 13075_2014_454_MOESM3_ESM.pdf]
